# TRPV1 activation and internalization is part of the LPS-induced inflammation in human iPSC-derived cardiomyocytes

**DOI:** 10.1038/s41598-021-93958-3

**Published:** 2021-07-19

**Authors:** Katherine Sattler, Ibrahim El-Battrawy, Lukas Cyganek, Siegfried Lang, Huan Lan, Xin Li, Zhihan Zhao, Jochen Utikal, Thomas Wieland, Martin Borggrefe, Xiaobo Zhou, Ibrahim Akin

**Affiliations:** 1 First Department of Medicine, Faculty of Medicine, University Medical Center Mannheim (UMM), University of Heidelberg, Theodor-Kutzer-Ufer 1-3, 68167 Mannheim, Germany; 2 European Center for AngioScience (ECAS), Mannheim, Germany; 3 DZHK (German Center for Cardiovascular Research), Partner Sites Heidelberg-Mannheim and Göttingen, Heidelberg, Göttingen, Germany; 4 Stem Cell Unit, Clinic for Cardiology and Pneumology, University Medical Center Göttingen, Göttingen, Germany; 5 Skin Cancer Unit, German Cancer Research Center (DKFZ), Heidelberg, Germany; 6 Department of Dermatology, Venereology and Allergology, University Medical Center Mannheim, University of Heidelberg, Mannheim, Germany; 7 Institute of Experimental and Clinical Pharmacology and Toxicology, Medical Faculty Mannheim, University of Heidelberg, Mannheim, Germany; 8 Key Laboratory of Medical Electrophysiology of Ministry of Education and Medical Electrophysiological Key Laboratory of Sichuan Province, Institute of Cardiovascular Research, Southwest Medical University, Luzhou, China

**Keywords:** Mechanisms of disease, Cardiovascular biology

## Abstract

The non-selective cation channel transient receptor potential vanilloid 1 (TRPV1) is expressed throughout the cardiovascular system. Recent evidence shows a role for TRPV1 in inflammatory processes. The role of TRPV1 for myocardial inflammation has not been established yet. Human induced pluripotent stem cell (iPSC)-derived cardiomyocytes (hiPSC-CM) from 4 healthy donors were incubated with lipopolysaccharides (LPS, 6 h), TRPV1 agonist capsaicin (CAP, 20 min) or the antagonist capsazepine (CPZ, 20 min). TRPV1 expression was studied by PCR and western blotting. TRPV1 internalization was analyzed by immunofluorescence. Interleukin-6 (IL-6) secretion and phosphorylation of JNK, p38 and ERK were determined by ELISA. TRPV1-associated ion channel current was measured by patch clamp. TRPV1-mRNA and -protein were expressed in hiPSC-CM. TRPV1 was localized in the plasma membrane. LPS significantly increased secretion of IL-6 by 2.3-fold, which was prevented by pre-incubation with CPZ. LPS induced TRPV1 internalization. Phosphorylation levels of ERK, p38 or JNK were not altered by TRPV1 stimulation or inhibition. LPS and IL-6 significantly lowered TRPV1-mediated ion channel current. TRPV1 mediates the LPS-induced inflammation in cardiomyocytes, associated with changes of cellular electrophysiology. LPS-induced inflammation results in TRPV1 internalization. Further studies have to examine the underlying pathways and the clinical relevance of these findings.

## Introduction

The non-selective cation channel transient receptor potential vanilloid 1 (TRPV1) mediates heat and pain sensation in various organ systems. The ion-channel is activated by a variety of chemical ligands^1^ or physical stimuli^2^. The receptor forms a homotetramer within the plasma membrane and is permeable for monovalent or divalent cations^3^. TRPV1 receptor was found primarily on sensory neurons but also in many different cell types^3,4^, being involved in neurogenic inflammation, pain, autoimmune diseases and glucose homeostasis^3–5^. Recently, the expression of TRPV1 on left ventricular cardiomyocytes and primary neonatal cardiomyocytes was demonstrated^6^. For the cardiovascular system, an ambiguous role of TRPV1 was described, as channel activation was associated with reperfusion injury^6,7^, but also vascular remodeling^8^, arterial and pulmonary hypertension^9,10^ and ventricular hypertrophy^1,11^.

Lipopolysaccharides (LPS) are well-known mediators of inflammation. As published previously, LPS induce an inflammatory response in human iPSC-derived cardiomyocytes, associated with the release of interleukin-6^12^. In trigeminal sensory neurons, a role of TRPV1 for LPS-induced inflammation has been demonstrated^13^. A functional link between TRPV1 and the LPS receptor, Toll-like receptor 4 (TLR4), was described recently in sensory neurons^14^, as was receptor internalization after stimulation with the agonist, capsaicin^15^. In the current study, we tested the hypothesis that TRPV1 takes part in LPS-mediated inflammation in cardiomyocytes by an interaction between TLR4 and TRPV1. We studied TRPV1 expression and the inflammatory response upon LPS incubation under the influence of TRPV1 agonists and antagonists in hiPSC-derived cardiomyocytes. In addition, channel localization was assessed by immunofluorescence staining, and TRPV1 ion channel current by patch clamp.

## Materials and methods

### Generation of human iPS cells (hiPSC)

Primary somatic cells were derived from skin biopsies or blood samples of four healthy donors after written informed consent had been obtained. The study was approved by the Ethics Committee of the Medical Faculty Mannheim (2009-350N-MA, 2018-565N-MA) and of the University Medical Center Göttingen (10/9/15), being in accordance with the Declaration of Helsinki. Human iPS-cell (hiPSC)-models were generated by the Stem Cell Unit (SCU) at the University Medical Center Göttingen (support DZHK and DFG SFB1002 S1). The hiPSC line of donor 1 (D1) was obtained by transfection of lentivirus particles^16^ and cell lines of donor 2, donor 3 and donor 5 (D2: UMGi014-B clone 1, D3: UMGi124-A clone 11, and D5: UMGi130-A clone 5) by use of integration free reprogramming methods^17,18^.

### Generation of human iPSC-derived cardiomyocytes

Differentiation of hiPSC into cardiomyocytes (hiPSC-CM) was performed under feeder free conditions^19^. hiPSC were grown in TeSR-E8 medium (#05990, Stemcell Technologies) and changed to RPMI 1640-Glutamax (#61870044, Gibco) containing sodium pyruvate (#11360039, Gibco), penicillin/streptomycin (#15140122, ThermoFisher), B27 (#17504001, Gibco) and ascorbic acid (#A8960, Sigma) after start of differentiation. The differentiation factors CHIR99021 (#130-103-926, StemMacs), BMP-4 (#314-BP-010, R&D Systems), activin A (#338-AC-010, R&D Systems), FGF-2 (#130-093-841, miltenyi biotec) and IWP-4 (#72552, Stemcell Technologies) were added at different time points. During the third week of differentiation, cardiomyocytes were selected by using a lactate-supplemented, glucose- and glutamine-free RPMI medium (#16-101-1A, Biological Industries). Afterwards, cells were fed with RPMI 1640-Glutamax as before. The number of experiments and of biological replicates in each experiment depended on the number of the availability of cells in culture wells showing 80% confluence at an age of ≥ 40 days of differentiation. As during the differentiation of hiPSC into cardiomyocytes a great variability of confluent cells develop, the preparation of even experimental groups was not always possible. However, in each experiment controls and replicates showing about 80% confluence at an age of ≥ 40 days of differentiation were included. Altogether, the experiments are based on twelve independent differentiation runs which were performed from November 2015 to August 2017 and from January 2019 to August 2020 using an identical cell culture protocol.

### RNA extraction, cDNA synthesis and RT-qPCR

hiPSC-CMs were lysed with RLT lysis buffer (#79216, Qiagen). RNA was extracted using the RNeasy MiniKit (#74106, Qiagen). Equal amounts of RNA were reversely transcribed to cDNA with oligo d(T)_15_ primers using AMV reverse transcriptase (#10109118001, Roche). Quantitative real time PCR was done with hot start Taq DNA-polymerase and SYBR-Green (#119405, Bioron) using commercially available primers (GAPDH, #PPH00150F; TNNT2, #PPH025619A; TRPV1, #PPH08086F, Qiagen). GAPDH was used as housekeeping gene to normalize expression. For each of the independent experiments, the mean CT value of 3 biological replicates was calculated from two technical replicates. Normalized mRNA expression was calculated by using ΔCT = (CT_gene of interest_ − CT_housekeeping gene_).

### Cell incubation studies

hiPSC-CMs at ≥ 40 days of differentiation were starved by using RPMI1640 medium (#11875093, ThermoFisher) without ascorbic acid and B27 for 24 h (h). TRPV1 antagonist capsazepine (CPZ, 100 µM, #12084, Sigma) or agonist capsaicin (CAP, 10 µM, #C191, Sigma) was added (20 min, 37 °C, 5% CO_2_). Lipopolysaccharides (LPS, #L2630, Sigma) were used at 1 µg/mL for 6 h, if not indicated otherwise (37 °C, 5% CO_2_). In some cases, CAP and CPZ were added prior to LPS (same concentrations and times as for single substance incubations). The TRPV2-specific antagonist gadolinium(III) chloride^20^ (#439770, Sigma) was used at 200 µM (20 min, 37 °C, 5% CO_2_). After incubation, supernatants and cells were harvested.

### Western blotting

Cells were lysed in RIPA buffer (#R0278, Sigma) supplemented with protease and phosphatase inhibitors (#P8340, #P5726, #P0044, Sigma). Protein concentration of lysates was determined by BCA assay (#23227, Thermo Scientific). 10 µg of proteins of cell lysates or TRPV1 control peptide (#ACC-030, alomone labs) were resolved in 10% acrylamide gels and transferred to PVDF membranes. Primary antibodies were incubated at 4 °C overnight (1:500, #TA336871, acris, or #ACC-030, alomone labs), followed by the secondary antibody (#A0545, Sigma, 1:5000, 1 h, room temperature (RT)). Signals were detected by chemiluminescence (#32209, Thermo scientific) using a LAS-1000 system (Fujifilm).

### MTT assay

The MTT assay measures the metabolic activity and viability of cells via NAD(P)H-dependent cellular oxidoreductase enzymes. Equal numbers of hiPSC-CMs seeded in a 96 well plate were analyzed with a commercially available MTT-kit according to manufacturer’s instructions (#PK-CA707-30006, PromoCell). Cells were incubated with capsazepine or capsaicin as described above. Blanks consisted of wells containing medium but no cells. Measurements of three independent experiments with a total n = 4–17 wells per reagent were included in the analysis.

### Enzyme-linked immunosorbent assays

Interleukin-6 in supernatants was measured with an enzyme-linked immunosorbent assay kit (#ELH-IL6-1, RayBio) according to manufacturer’s instructions. The measurements of each of the 3 to 17 independent experiments were done in duplicate.

The extent of phosphorylation of ERK, JNK and p38 was determined with a cell-based enzyme-linked immunosorbent assay kit (#CBEL-ERK-SK, RayBio) according to manufacturer’s instructions. Measurements were done in two to three biological replicates in three independent experiments. The mean optical density of the background, determined by the optical density of cells treated with agonist or antagonist but without application of antibody, was subtracted from the respective results of each treatment group.

### Immunofluorescence

hiPSC-CMs were grown on culture slides (#354114, Falcon) for at least 5 days.

Localization of TRPV1 and quantification of TRPV1 internalization were assessed by the same immunofluorescence protocol. Cells were incubated with the agonist capsaicin (CAP) or the antagonist capsazepine (CPZ), prior to incubation with LPS, as stated above. After incubation with anti-TRPV1 primary antibody (1:50, 1 h, 37 °C, #ACC-030, alomone labs) and anti-rabbit secondary antibody (1:100, 1 h, RT, FITC-coupled: #FI-1000, Vector laboratories; PE-coupled: #abin2145659, antibodies online), intact cardiomyocytes were identified by incubation with wheat germ agglutinate plasma membrane antibody-Alexa350 (1%, 30 min, 37 °C, #WM263, Thermo Fisher), followed by fixation with 4% formaldehyde (5 min), permeabilization with Triton 0.5% (5 min), incubation with Alexa647-anti troponin T (1:50, 1 h, RT, #565744, BDSciences), and nuclear staining with propidiumjodide (#H-1300, Vector laboratories). For fluorescence quantification, Image J (v 1.52a, NIH) was used to determine corrected total cellular fluorescence (CTCF). CTCF was obtained by outlining each cell and measuring the cell area and the mean fluorescence of each fluorochrome. Additional measurements of several background readings per slide were then combined into the formula: Corrected total cellular fluorescence (CTCF) = integrated density − (area of selected cell × mean fluorescence of background readings) per cell^21^. CTCF was normalized to cell area to compensate for the high variety of cell area values. Microscopy was performed with a Leica DMRE microscope (Leica Application Suite V4.4.0, Leica Microsystems CMS). ImageJ (v 1.52a, NIH) was used to color single images of multicolor merged images for presentation purpose.

### Patch clamp

Standard patch-clamp recording techniques were applied as described before^12^. To isolate the TRPV1 ion channel current, specific channel blocker capsazepine (100 µM) was used, and the blocker-sensitive currents were analyzed. The bath solution for TRPV1 current measurements contained [mmol/L]: 140 NaCl, 5.4 TEA-Cl, 2.0 CaCl_2_, 1.0 MgCl_2_, 10 HEPES, 10 glucose, pH 7.4 (CsOH). The pipette solution contained [mmol/L]: 135 CsCl, 0.1 CaCl_2_, 10 EGTA, 1.0 MgATP, 1.0 MgCl_2_, 10 HEPES, 0.1 NaGTP, pH 7.4 (CsOH).

### Statistics

Data are shown as mean ± standard deviation, median (minimum/maximum), or number (percent). Group comparisons were performed by t-test or Mann–Whitney Rank Sum test, depending on distribution of data. Multiple groups were compared by Kruskal–Wallis-test, followed by Dunn’s multiple comparison post-hoc test. For the analysis of intracellular signaling pathways the mean + 2 standard deviations (mean + 2SD) method was applied to discriminate signals from background noise^22^. *p* values are understood to be strictly descriptive. Statistical significance was assumed for *p* < 0.05 (two-tailed). The calculations were performed and the graphs were drawn with InStat (GraphPad Software, San Diego, USA) and SPSS (IBM SPSS Software GmbH, Munich).

### Ethics approval and consent to participate

The study was approved by the Ethics Committee of the Medical Faculty Mannheim, University of Heidelberg (2009-350N-MA) and by the Ethics Committee of University Medical Center Göttingen (approval number: 10/9/15), and carried out in accordance with the approved guidelines. Written informed consent was obtained from all participants or their legal representatives.

## Results

### TRPV1 is present in human iPSC-derived cardiomyocytes

**Figure 1:**
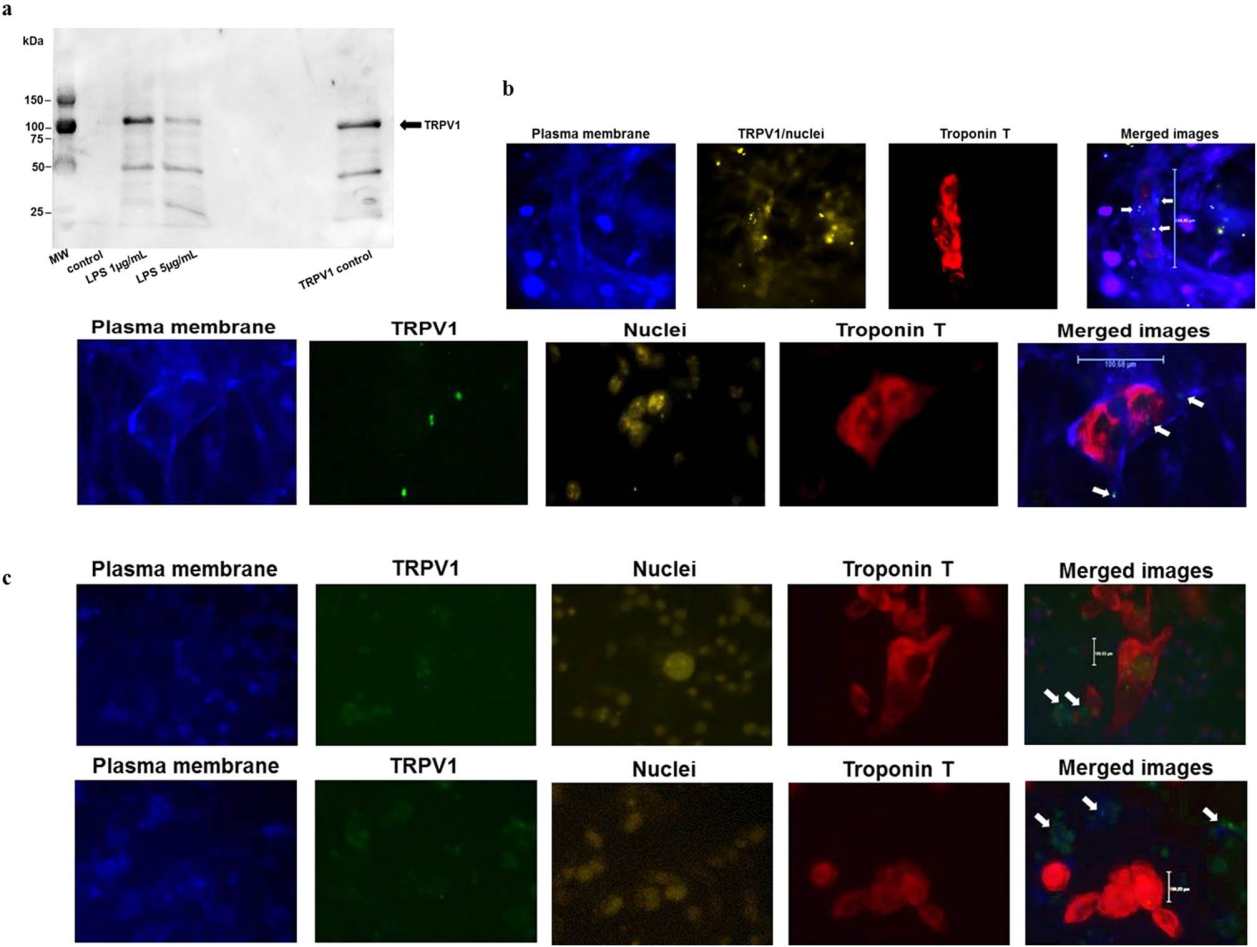
(**a**) Detection of TRPV1 protein in hiPSC-cardiomyocytes by western blotting. Cells were incubated with LPS for 6 h, 37 °C. Unstimulated cells served as a control (“control”). TRPV1 control protein was loaded at equal concentration. Primary antibody: rabbit polyclonal anti-TRPV1, #ACC-030, alomone labs, 1:500, 4 °C overnight. TRPV1 control: TRPV1 control peptide, #ACC-030, alomone. kDa—kiloDalton, MW—molecular weight marker. A representative experiment out of 4 independent experiments is shown. (**b**) TRPV1 is present in the plasma membrane of hiPSC-cardiomyocytes (troponin T-positive cells). Cells were stained by immunofluorescence for plasma membrane, TRPV1, troponin T and nuclei (upper panel: Alexa350-wheat germ agglutinate anti-plasma membrane, anti-TRPV1 primary/PE-anti-rabbit secondary antibody, Alexa647-anti troponin T, propidiumjodide. Lower panel: Alexa350-wheat germ agglutinate plasma membrane, anti-TRPV1 primary/ FITC-anti-rabbit secondary antibody, Alexa647-anti troponin T, propidiumjodide). Arrows in the merged images denote TRPV1. Magnification was 40x. Scale bars: Upper panel: 244.45 µm. Lower panel: 100.68 µm. Independent experiments, n = 3. Per experiment, images of 6–16 different positions were taken. (**c**) TRPV1 is present in the plasma membrane of cell types other than cardiomyocytes (troponin T-negative cells). Cells were stained by immunofluorescence as in Figure (**b**), lower panel. Arrows in the merged images denote TRPV1 in cells other than hiPSC-CMs. Magnification was 40 × . Scale bars: 100.03 µm. Independent experiments, n = 3. Per experiment, images of 6–16 different positions were taken.

The presence of TRPV1 in human iPSC-derived cardiomyocytes (hiPSC-CM) was tested by RT-qPCR and by western blotting. Both mRNA and protein of TRPV1 were found in cell lysates*.* The PCR of TRPV1-mRNA expression showed CT values between 26.48 and 36.12 (moderate to low expression), and, after normalization with GAPDH, ΔCT values (CT_TRPV_-CT_GAPDH_) of 10.06 to 11.05 (Table 1). Western blotting with two different antibodies detected the TRPV1 band at 100 kDa (Fig. 1a and supplemental Fig. 1). Localization studies by immunofluorescence showed the channel within the plasma membrane of hiPSC-CMs (Fig. 1b), but also in other, undefined cell types of the cell culture negative for troponin T (Fig. 1c).

**Table 1:** TRPV1 mRNA expression in hiPSC-derived cardiomyocytes.

	PCR 1	PCR 2	PCR 3	Mean ± SD, all experiments
TRPV1 mRNA expression^a^	26.48	36.20	26.49	29.72 ± 5.61
GAPDH mRNA expression^a^	16.42	25.71	15.44	19.19 ± 5.67
TRPV1 expression normalized to GAPDH expression^b^	10.06	10.49	11.06	10.53 ± 0.50

Results of three independent experiments (PCR 1–3) of quantitative real time-PCRs. GAPDH expression was used as expression of a housekeeping gene for normalization.

^a^Mean CT value of three technical replicates.

^b^ΔCT = CT_TRPV1_ − CT_GAPDH_; SD, standard deviation.

### Inhibition of TRPV1 enhances cell metabolism

**Figure 2:**
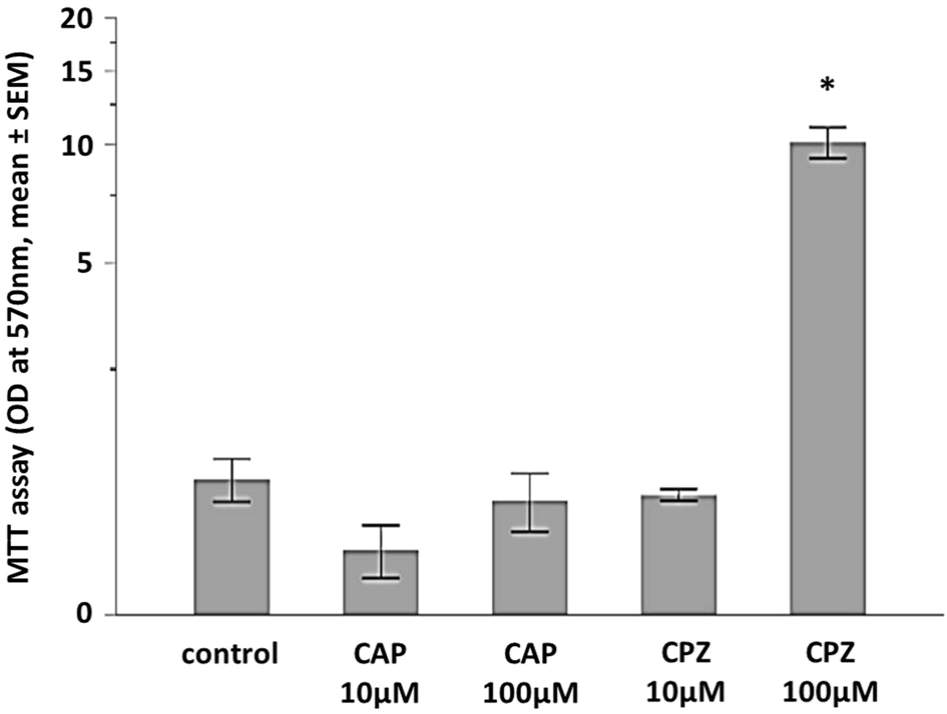
The metabolic activity of hiPSC-CMs is not affected by capsaicine (CAP), but by capsazepine (CPZ). Presented is the normalized optical density (OD) at 570 nm after application of a MTT assay measuring metabolic activity via NAD(P)H-dependent cellular oxidoreductase enzymes (mean ± standard error of the mean (SEM)). Kruskal–Wallis *p* < 0.0001, *p < 0.001 CPZ 100 µM versus control. For better presentation of low values, the y-axis was scaled logarithmic. Unstimulated cells, n = 16, n = 3 independent experiments, n = 3–7 biological replicates per experiment. CAP 10 µM, n = 9, n = 2 independent experiments, n = 3 and 6 biological replicates per experiment. CAP 100 µM, n = 14, n = 3 independent experiments, n = 3–6 biological replicates per experiment. CPZ 10 µM, n = 4, n = 1 independent experiment, n = 4 biological replicates. CPZ 100 µM, n = 17, n = 3 independent experiments, n = 3–9 biological replicates per experiment.

As the TRPV1 agonist capsaicin was shown to induce time- and dose-dependently cell death in different cell types^23–25^, we tested the effects of capsaicin (CAP) and the TRPV1 antagonist, capsazepine (CPZ), on hiPSC-cardiomyocyte viability by the MTT assay measuring metabolic activity via NAD(P)H-dependent cellular oxidoreductase enzymes (MTT clearance). Stimulation of cells with CAP used at 10 µM for 20 min had no effect on cellular metabolism (Fig. 2). Increasing the CAP concentration to 100 µM slightly increased, but not decreased MTT clearance. In contrast, incubation with 100 µM capsazepine for 20 min clearly increased the MTT clearance (*p* < 0.001). The cellular reaction upon CPZ was dose dependent, as a concentration of 10 µM did not have a significant effect (Fig. 2). For chloroform or methanol, the solvents of CAP and CPZ, respectively, no effects on cellular metabolism were observed when used in the same concentration (data not shown).

### LPS induce inflammation in human iPSC-derived cardiomyocytes

**Figure 3:**
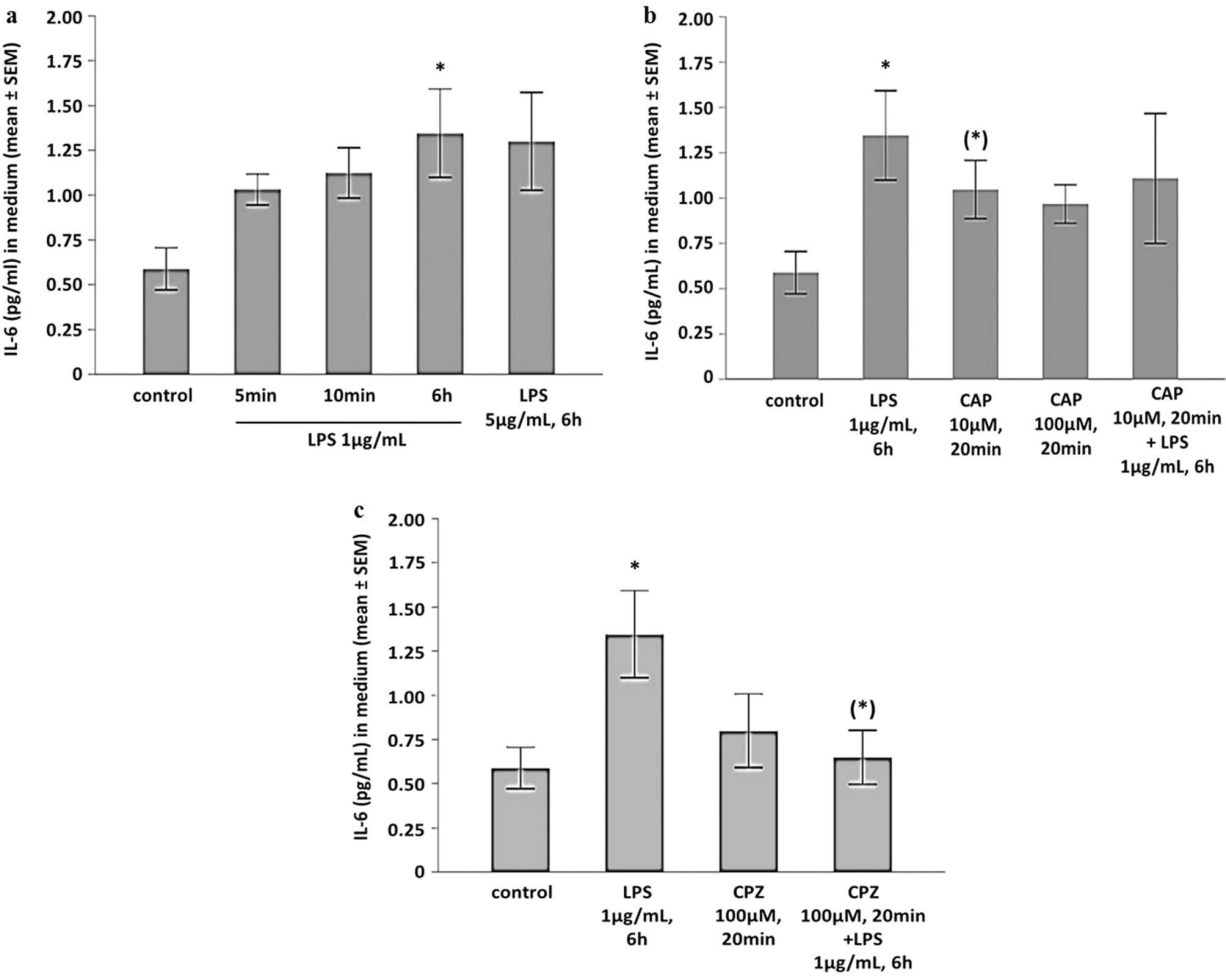
(**a**) LPS induces the release of interleukin-6 into the cell culture medium (mean ± standard error of the mean (SEM)). Kruskal–Wallis *p* = 0.0136; *p < 0.01 LPS 1 µg/mL, 6 h, versus unstimulated controls. Unstimulated cells, n = 16, n = 5 independent experiments, n = 1–5 biological replicates per experiment. LPS 1 µg/mL, 6 h, n = 15, n = 5 independent experiments, n = 1–7 biological replicates per experiment. LPS 1 µg/mL, 5 or 10 min, n = 3, n = 1 independent experiment, n = 3 biological replicates. LPS 5 µg/mL, n = 5, n = 4 independent experiments, n = 1–2 biological replicates per experiment. (**b**) Capsaicin (CAP, 10 µM, 20 min) mediates a non-significant release of interleukin-6 which is lower than the LPS-induced interleukin-6 production (LPS, 1 µg/mL, 6 h). Capsaicin (100 µM, 5 min) does not have an effect on interleukin-6 release. Presented is the mean ± standard error of the mean (SEM). Kruskal–Wallis *p* = 0.037; * *p* < 0.05 LPS versus unstimulated controls; (*) *p* < 0.1 CAP 10 µM versus unstimulated controls. Unstimulated cells, n = 16, n = 5 independent experiments, n = 1–5 biological replicates per experiment. LPS 1 µg/mL, 6 h, n = 15, n = 5 independent experiments, n = 1–7 biological replicates per experiment. CAP 10 µM, n = 9, n = 4 independent experiments, n = 1–5 biological replicates per experiment. CAP 100 µM, n = 3, n = 1 independent experiment, n = 3 biological replicates. CAP + LPS, n = 5, n = 3 independent experiments, n = 1–3 biological replicates per experiment. (**c**) Capsazepine (CPZ, 100 µM, 20 min) prevents LPS-induced interleukin-6 secretion. Presented is the mean ± standard error of the mean (SEM). Kruskal–Wallis *p* = 0.017; *p < 0.01 LPS versus unstimulated controls, (*) *p* < 0.1 CPZ + LPS versus LPS. Unstimulated cells, n = 16, n = 5 independent experiments, n = 1–5 biological replicates per experiment. LPS 1 µg/mL, 6 h, n = 15, n = 5 independent experiments, n = 1–7 biological replicates per experiment. CPZ, n = 6, n = 4 independent experiments, n = 1–2 biological replicates per experiment. CPZ + LPS, n = 7, n = 4 independent experiments, n = 1–4 biological replicates per experiment.

Incubation of hiPSC-CMs with LPS (1 µg/mL for 6 h) stably induced the secretion of interleukin-6 (IL-6) into the cell medium (Fig. 3a, p < 0.01 vs. unstimulated controls). As a time-dose relationship was described for activation of TRPV1^15^, we tested whether this relationship was present in hiPSC-CMs as well. We found that even short-time incubation with LPS (5 or 10 min) induced an increase of IL-6 secretion, as did a higher LPS concentration (5 µg/mL, 6 h; Fig. 3a).

### Pre-activation of TRPV1 attenuates IL-6 release

To test whether activation of TRPV1 per se would stimulate IL-6 secretion, hiPSC-CMs were incubated with the TRPV1-agonist capsaicin (CAP, 10 µM, 20 min, and 100 µM, 5 min). Incubation with 10 µM for 20 min induced the secretion of IL-6 to a non-significant extent (*p* < 0.1; Fig. 3b). As recently published, receptor internalization and degradation was observed after 10 min of agonist incubation^15^. Short time incubation for only 5 min with a five-time higher concentration of capsaicin (CAP 100 µM) did not induce a pro-inflammatory phenotype (Fig. 3b). When pre-incubated with capsaicin (10 µM, 20 min), the extent of the inflammatory effect of LPS was reduced (Fig. 3b).

### TRPV1 mediates LPS-induced inflammation

hiPSC-CMs were pre-incubated with capsazepine (CPZ, 100 µM) for 20 min prior to LPS incubation (1 µg/mL, 6 h). While CPZ alone had no effect, pre-incubation with capsazepine prevented LPS-induced IL-6 secretion (Fig. 3c). In contrast, pretreatment with the TRPV2-antagonist gadolinium(III) chloride (GadCl_3_, 200 µM, 20 min) showed only a trend towards reduction of LPS-mediated inflammation (ANOVA *p* = 0.07, data not shown).

### Analysis of intracellular signaling pathways following TRPV1 stimulation

A multitude of pathways mediates the intracellular response uponTRPV1 activation or inhibition. These pathways are not defined in cardiomyocytes yet. We studied the activation of the JNK, ERK and p38 pathways by determining the phosphorylation of these enzymes in hiPSC-CM incubated with CAP, CPZ, or with LPS, and in cells incubated with CAP or CPZ and subsequently with LPS (1 µg/mL, 6 h). Interestingly, we could not observe any changes in the amount of unphosphorylated or phosphorylated JNK, ERK or p38, but found phosphorylation levels corresponding to the background signal (Table 2). Thus, TRPV1-mediated LPS-induced inflammation and CAP-mediated inflammation do not seem to depend on JNK-, ERK- or p38-phosphorylation in hiPSC-cardiomyocytes.

**Table 2:** Intracellular pathway signaling by phosphorylation of JNK, ERK and p38 after TRPV1 stimulation or inhibition in hiPSC-derived cardiomyocytes.

	Control	CAP	CPZ	LPS	CAP + LPS	CPZ + LPS
JNK	0.75 ± 0.56	0.03 ± 0.50	− 0.35 ± 0.65	− 0.28 ± 1.0	0.03 ± 0.79	0.54 ± 0.50
Phospho-JNK	1.44 ± 1.10	1.11 ± 1.11	1.14 ± 0.99	0.84 ± 1.27	0.87 ± 1.38	1.69 ± 0.58
ERK	0.38 ± 0.86	− 0.08 ± 0.55	− 0.03 ± 0.56	0.04 ± 0.33	0.11 ± 0.72	0.21 ± 0.35
Phospho-ERK	0.23 ± 0.82	0.52 ± 0.21	0.21 ± 0.53	0.01 ± 0.47	0.32 ± 0.24	1.07 ± 0.69
p38	− 0.07 ± 0.89	0.34 ± 0.39	− 0.23 ± 0.86	− 0.22 ± 0.69	− 0.28 ± 1.15	0.91 ± 0.35
Phospho-p38	− 0.38 ± 0.58	− 0.74 ± 0.77	0.33 ± 1.17	− 0.21 ± 0.58	− 1.07 ± 1.31	0.76 ± 0.76

Results of optical density measurements.

Optical density (OD) of unphosphorylated and phosphorylated enzymes after background signal subtraction as measured by ELISA. The background signal was measured in wells treated with the respective reagent but not exposed to the respective antibody. Results are derived from three independent experiments consisting of two to three biological replicates per experiment. As the mean + 2SD method showed all signals within background levels, no statistical comparison test was applied to the data. LPS, 1 µg/mL, 6 h, CAP, 10 µM, 20 min, CPZ, 100 µM, 20 min. CAP—capsaicin; CPZ—capsazepine; LPS—lipopolysaccharides.

### TRPV1 is internalized upon stimulation with LPS

**Figure 4:**
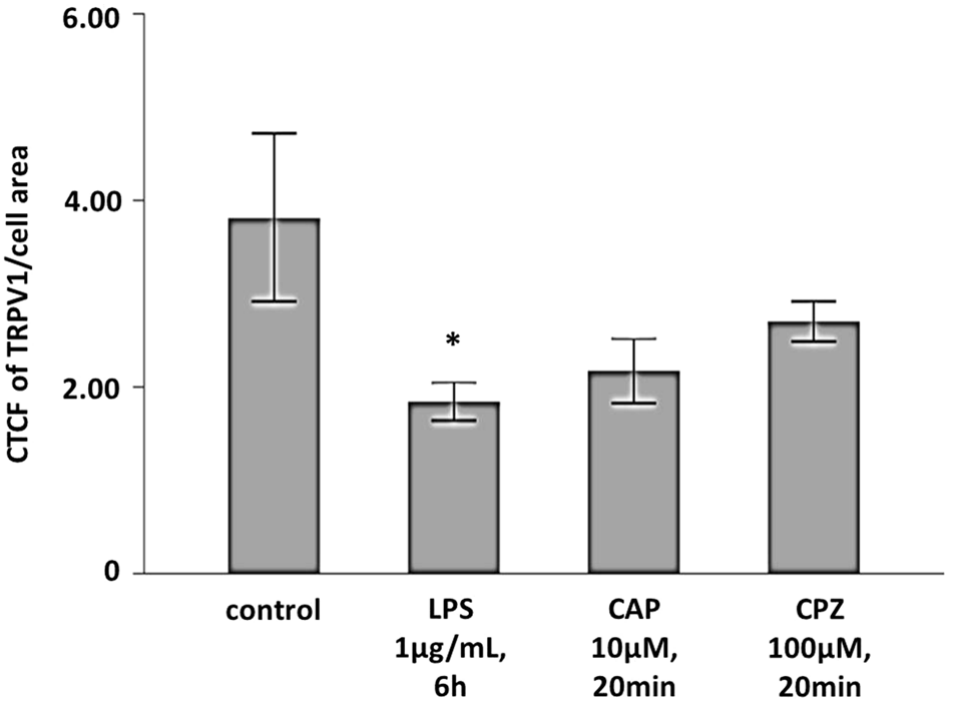
TRPV1 is internalized upon stimulation with LPS, as determined by corrected total cellular fluorescence (CTCF) of the fluorochrome-marked TRPV1 channel. Presented is the mean ± standard error of the mean (SEM). Kruskal–Wallis *p* = 0.005; *p < 0.05 unstimulated controls versus LPS. Results are derived from two independent experiments. Unstimulated cells, n = 26; n = 8 and n = 18 biological replicates per experiment. LPS (1 µg/mL, 6 h), n = 36; n = 12 and n = 24 biological replicates per experiment. CAP + LPS (CAP 10 µM, 20 min, + LPS, 1 µg/mL, 6 h), n = 29; n = 13 and n = 16 biological replicates per experiment. CPZ + LPS (CPZ, 100 µM, 20 min, + LPS, 1 µg/mL, 6 h), n = 39; n = 9 and n = 30 biological replicates per experiment.

Upon activation by an agonist, TRPV1 becomes desensitized either by phosphorylation and/or by internalization and degradation, as shown for DRG-neurons and HEK293 cells^15^. We therefore hypothesized that the exposure of hiPSC-CMs to LPS would lead to TRPV1 internalization on these cells as well. Indeed, analysis of the fluorescence intensity of TRPV1 using immunofluorescence demonstrated reduction of the TRPV1 channel density after incubation with LPS (1 µg/mL, 6 h; Fig. 4).

### Long-term inflammation leads to prolonged impairment of TRPV1 ion channel current

**Figure 5:**
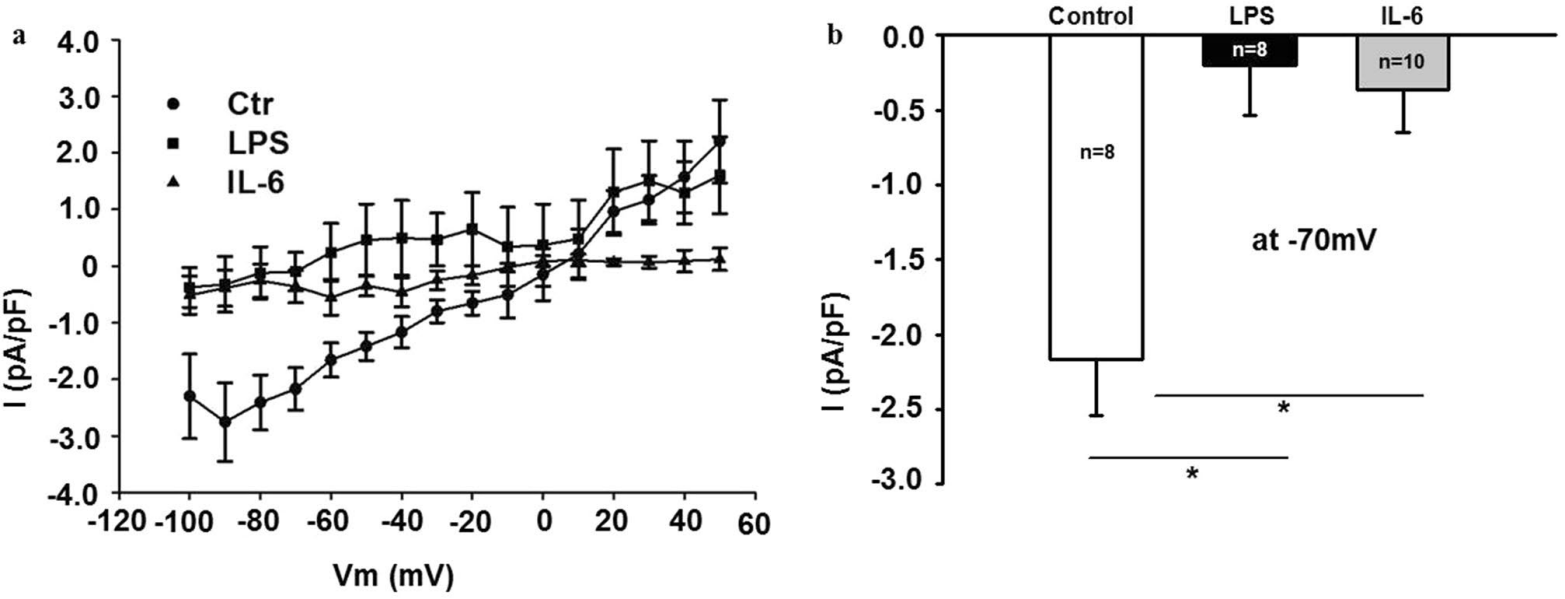
TRPV1 ion channel current is attenuated by LPS and by interleukin-6 (IL-6). (**a**) I–V curves of the capsazepine-sensitive TRPV1 current in absence (control, Ctr, circles) and presence of LPS or IL-6 (LPS, 1 µg/mL, 6 h: squares, IL-6, 10 ng/mL, 6 h: triangles). Vm—membrane potential. (**b**) Mean values of the capsazepine-sensitive TRPV1 current at − 70 mV in absence (control, Ctr) and presence of LPS or IL-6 (LPS, 1 µg/mL, 6 h. IL-6, 10 ng/mL, 6 h). *p < 0.05. Data of two independent experiments are shown. Unstimulated control cells, n = 8; n = 4 biological replicates per experiment. LPS, n = 8; n = 4 biological replicates per experiment. IL-6, n = 10; n = 5 biological replicates per experiment.

Prolonged TRPV1 activation by an agonist leads to the reduction of calcium influx via the channel, paralleled by the degradation of the channel^15^. We therefore tested whether prolonged LPS incubation would alter TRPV1-associated cellular electrophysiology. Indeed, incubation of hiPSC-CMs with LPS (1 µg/mL, 6 h) reduced the TRPV1-associated current (Fig. 5a, b). To discern whether electrical receptor activity would be attenuated specifically by exposure to LPS or in general by an inflammatory environment, cells were incubated with interleukin-6 (10 ng/mL, 6 h). The generation of inflammation by this cytokine reduced the TRPV1 ion channel current to levels of LPS mediated reduction (Fig. 5a, b).

## Discussion

In the current study, we showed that hiPSC-derived cardiomyocytes express the TRPV1 channel within the plasma membrane, that TRPV1 mediates the LPS-induced inflammation, and that stimulation with LPS leads to TRPV1 internalization. In addition, we found a prolonged disturbance of TRPV-associated current induced by exposure to LPS, but also to the inflammatory cytokine interleukin-6.

TRPV1 is known for its role as a mediator of pain and heat sensation^3^. Its endogenous ligands are protons, ATP, anandamide, dopamines and lipoxygenase products^26^. Structure analyses show that the channel forms a homotetramer, consisting of six transmebrane domains with intracellular localization of both C- and N-terminus. Ligand binding takes place intracellulary with the exception of proton binding^3^. TRPV1 mediates many different effects in the cardiovascular system, being both protective and harmful, depending on the precise setting: TRPV1 was shown to mediate ventricular hypertrophy and to aggravate arterial hypertension and pulmonary hypertension, but on the other side to protect the myocardium from reperfusion injury^27^. In different organ systems, the participation of TRPV1 in inflammatory processes has become apparent, for example in inflammatory bowel diseases^28^ or neurogenic inflammatory diseases^26^. The precise role of TRPV1 for regular cardiomyocyte function is not established so far, but the presence of TRPV1 in cardiac muscle, more precisely, at costameres, intercalated discs and z-discs of cardiomyocytes, was demonstrated recently^29,30^. There are discrepant findings regarding the question of the localization of TRPV1 throughout the cardiovascular system: While the channel was identified in human vascular endothelial cells^31^, human aortic endothelial cells^32^ and vascular smooth muscle cells^10^, the localization within cardiac tissue is less clear. Hong et al. did whole murine heart staining by GFP-marked TRPV1. They did not detect any trace of the channel in atrial or ventricular cardiomyocytes, but found instead expression in nerve endings and endothelial cells^33^. However, Pei et al. and Hurt et al. found TRPV1 by western blotting and by immunostaining in primary mouse and rat cardiomyocytes^6,34^. Furthermore, TRPV1 localization was demonstrated in murine embryonic stem cells (ESC) and in ESC-derived cardiomyocytes^35,36^. While the expression of TRPV1 mRNA in iPSC and unspecified human iPSC-derived cardiac cells was shown recently^37^, to our knowledge our study is the first to describe the expression and localization of the channel protein in hiPSC-CMs. By immunofluorescence, we found TRPV1 present in the plasma membrane of hiPSC-CMs. As only isolated cultured cells were examined, we cannot ascertain the location of the channel in interconnecting cellular structures, such as myofibrils. The spatial distribution of the channel and its function in cellular networks has to be determined yet. The reason for the discrepant findings of TRPV1 localization is unclear so far. One could assume that in the whole heart TRPV1 is expressed primarily by endothelial cells and nerve endings, but is suppressed in cardiomyocytes by the interplay of signals from blood vessels or nerves. In this model, cardiomyocytes kept isolated in cell culture without the possibility to interact with different cardiac and non-cardiac cell types would regain their ability of TRPV1 expression and independent activation. Obviously, the statement of Yao et al. regarding vascular endothelial cells that “the expression pattern of TRP channels may change in different culture conditions and/or during serial passages of cultured cells. Therefore, data collected from cultured cell lines may not truly reflect the in vivo expression pattern in the native tissues” holds true for cardiac TRPV1 expression as well^38^. Further work on cardiac TRPV1 function and expression is needed prior to carrying results of cellular TRPV1 studies into whole organisms.

The precise role of TRPV1 for regular cardiomyocyte function is not established so far. Only one study examined the role of TRPV1 on hiPSC-derived cardiomyocytes, finding increased growth upon stimulation by heat or stimulation with a TRPV1 agonist^37^. We used hiPSC-CMs to study TRPV1 function, as this cell type is well established in our laboratory^12,39,40^. We found that the TRPV1 agonist capsaicin (CAP) stimulated cells to produce a slight increase of interleukin-6 secretion, while the inflammatory mediator LPS induced time-dependently a significant production of IL-6. A complete reversal of the LPS-induced IL-6 release was observed after pre-treatment with the TRPV1 antagonist capsazepine (CPZ). As the TRPV2 antagonist gadolinium(III) chloride was able to attenuate the LPS effect as well, the LPS effect seems to rely only partially on the activation of TRPV1. Interestingly, however, similar results were observed in different settings before^41,42^. Whether a functional TRPV1 channel tetramer is required for the interaction with LPS, whether LPS binds directly to the channel, or whether a functional link between TRPV1 and the LPS receptors exists is unclear so far, as is the time of channel recycling back into the plasma membrane. As pre-incubation with the agonist capsaicin attenuated the LPS-induced IL6-release but did not itself induce IL-6, a functional link between TRPV1 and the LPS receptors seems likely. Many different intracellular pathways are described following the activation of TRPV1: p44/42 MAPK^43^, PKC/CRB^44^ or mTOR^45^ in skeletal muscle, PI3K/Akt/eNOS/NO in endothelial cells^46^, or PKA activation in nociceptive neurons^47^. In our model, we did not find an involvement of the phosphorylation of JNK, ERK or p38 in TRPV1 signaling. Recently, the increase of phosphorylation of ERKs and p38 was described in H9C2 cells after incubation with capsaicin for 24 to 48 h^1^. For short term exposure of cardiomyocytes to agonists or antagonists of TRPV1, as in our study, the pathways mediating the TRPV1 effect have to been defined yet.

LPS is known to affect the conductance of different ion-channels in cardiomyocytes^12^. Recently, an increase of calcium inward current via TRPV1 by LPS stimulation was demonstrated^13^, and TRPV1-sensitization by short term incubation with LPS or paclitaxel was demonstrated in neurons^13,14^. However, we chose different experimental settings compared to the previously published studies: Instead of studying receptor stimulation by an agonist (i.e. capsaicin) after short term incubation with LPS, we performed long-term LPS incubation to study its effect on “downstream” TRPV1 localization and function to closer model an ongoing inflammatory disease process. In addition, for the patch clamp studies we chose to incubate the cells with the antagonist capsazepine, in contrast to other studies which have used the agonist capsaicin^14,48^ for identifying the TRPV1-associated ion channel current. By doing so, we tried to prevent an effect on the membrane localization of the ion channel. The finding of the persistence of an impairment of TRPV1-associated current during prolonged LPS stimulation might correlate to a long-term perturbance of the channel during clinically relevant inflammatory processes. Interestingly, incubation with the pro-inflammatory cytokine interleukin-6 attenuated the channel current as well. This is an important extension to the finding of Yoshie et al.^49^ who demonstrated that TRPV1 localized in cardiac afferent nerves is related to ventricular arrhythmias after myocardial infarction, as we demonstrate that on cellular levels the TRPV1 channel has electrical function as well. Another interesting finding of our study is the effect of capsazepine on the metabolic activity of hiPSC-CMs. As the cells are not proliferating within the duration of an MTT assay, the results point to an increase of the metabolic state of hiPSC-CMs by TRPV1-inhibition by CPZ. CPZ acts by preventing (agonist)-induced receptor internalization^15^, but does not induce receptor internalization, as shown in our study. As the cells in the MTT assay were not pre-incubated with a TRPV1 agonist, capsazepine seems to have distinct effects on TRPV1 signaling as well, or might have TRPV1-independent effects which has been already described for cancer cell lines^50,51^. In any case, the finding warrants future studies addressing the participation of TRPV1 in cardiomyocyte metabolism. There are altogether conflicting results as to whether TRPV1 activation protects or endangers the cardiovascular system. TRPV1 signaling prevented from pressure overload-induced cardiac hypertrophy^52^, hypoxic injury^53^ and ischemia/reperfusion injury^54^. TRPV1 activation protected from atherosclerosis by eNOS production and induced angiogenesis^32^. On the other hand, TRPV1 signaling conferred the damage of cigarette smoke on cardiomyocytes^34^ and mediated the endothelial dysfunction induced by 12(S)-HETE in diabetic mice^55^. It is difficult to merge these different findings into one picture. Both in vitro and in vivo results clearly seem to depend on the respective experimental setting. Clearly, further information about the distribution of TRPV1 within a “living” cardiovascular system and the balancing factors of its in vivo expression are needed. In particular, the interplay of TRPV1 on different cell types connected with each other has to be studied in the future.

Our findings might have impact on several clinical aspects, as cardiac dysfunction caused by systemic inflammation or septic conditions has a prevalence of 13% to 60%^56^. Several derangements of different cellular mechanisms and functions have been described promoting sepsis-related cardiomyopathy^57,58^. We demonstrate that TRPV1 has a significant function for myocardial injury imposed by bacterial infection. The observation of the interference of an agonist with the effects of a pro-inflammatory agent might become important in the future, as capsaicin-derived compounds are already being used for treatment of neurogenic inflammatory pain in cutaneous diseases, headaches or neurogenic diseases^26^. Further studies are needed to specify the role of TRPV1 agonists or antagonists for immunomodulation in the clinical setting and to study their long-term side effects on the cardiovascular system. Indeed, capsaicin aggravated the effect of hypoxia/reoxygenation in a model using H9C2 cells^7^, while in different cell models, capsaicin was even shown to induce time- and dose-dependently cell death^23–25,59^. Our study provides a base for future projects targeting the effect of endogenous (i.e. endovanilloids, lipids) or exogenous (i.e. plant or animal toxins, camphor) ligands on cardiomyocytes and ultimately, on myocardial function^60^.

## Summary

This is the first study to show functional TRPV1 expression by hiPSC-derived cardiomyocytes and its participation in LPS-induced inflammation. This study is therefore an important extension of the studies performed in cardiomyocytes isolated from heart tissue^6^, as hiPSC-CMs provide a platform to study drug effects and pathophysiologic pathways. The results of this study point to a role of TRPV1 for cardiomyocyte electrophysiology during inflammation as well, in that this channel might be part of inflammation-associated arrhythmia^61,62^. Further studies are needed to define the related intracellular mechanisms, the interaction of the TRPV1 channel on cardiomyocytes with adjacent cardiac and non-cardiac cell types, and the relevance of these findings for clinical cardiology.

## Limitations

As the hiPSC-CMs grow in a mixed culture consisting of cardiomyocytes and primarily fibroblasts, we cannot rule out that inflammation was induced in non-cardiomyocytes as well, leading to the secretion of interleukin-6 by other cell types. Indeed, we demonstrated TRPV1 expression in hiPSC-CMs but also in cells other than hiPSC-CMs. Nevertheless, hiPSC-CMs have been used as a valuable model for inflammatory myocarditis before^63^, and the mixed culture might reflect cellular distribution in heart tissue more correctly than a cardiomyocyte-only culture. As with any research, increasing the number of replicates or expanding the methodology might have brought additional results to our study. However, we believe that the results presented might constitute the base for further research and discussion of cardiomyocyte physiology.

## Supplementary Information

Supplementary Figure 1.

## Data Availability

The datasets used and/or analyzed during the current study are available from the corresponding author on reasonable request.
