# Supplementary material for: TRPV1 activation and internalization is part of the LPS-induced inflammation in human iPSC-derived cardiomyocytes
Source: Sci Rep. 2021 Jul 19;11:14689. doi: 10.1038/s41598-021-93958-3 (PMC8289830; doi:10.1038/s41598-021-93958-3)

TRPV1 activation and internalization is part of the LPS-induced inflammation in human iPSC-derived cardiomyocytes

Katherine Sattler[1], Ibrahim El-Battrawy[1,2], Lukas Cyganek[2,3], Siegfried Lang[1,2], Jochen Utikal[4], Thomas Wieland[2,5], Martin Borggrefe[1,2], Xiaobo Zhou[1,2,6], Ibrahim Akin[1,2]

[1]First Department of Medicine, Faculty of Medicine, University Medical Centre Mannheim (UMM), University of Heidelberg, Mannheim, Germany, and European Center for AngioScience (ECAS),

[2]DZHK (German Center for Cardiovascular Research), Partner Site, Heidelberg-Mannheim and Göttingen, Germany

[3]Stem Cell Unit, Clinic for Cardiology and Pneumology, University Medical Center Göttingen, Göttingen, Germany

[4]Skin Cancer Unit, German Cancer Research Center (DKFZ), Heidelberg and Department of Dermatology, Venereology and Allergology, University Medical Center Mannheim, University of Heidelberg, Mannheim, Germany

[5] Institute of Experimental and Clinical Pharmacology and Toxicology, Medical Faculty Mannheim, University of Heidelberg, Mannheim, Germany

[6] Key Laboratory of Medical Electrophysiology of Ministry of Education and Medical Electrophysiological Key Laboratory of Sichuan Province, Institute of Cardiovascular Research, Southwest Medical University, Luzhou, China

Corresponding author:

Katherine Sattler, MD
1st Department of Medicine
University Medical Centre Mannheim (UMM)
Theodor-Kutzer-Ufer 1-3
68167 Mannheim, Germany
Phone: 0049-6201-383-2512
Fax: 0049-6201-383-2012
e-mail: Katherine.Sattler@umm.de

Figure legends.

Supplemental Figure 1. Detection of TRPV1 protein in hiPSC-cardiomyocytes by western blotting with a different antibody than used in Figure 1. Cells were incubated with 1µg/mL or 5µg/mL of LPS for 6 h, 37°C. Unstimulated cells served as a control ("control"). TRPV1 control protein was loaded at equal protein concentration. Primary antibody: rabbit polyclonal anti-TRPV1, #TA336871, acris, 1:500, 4°C overnight. TRPV1 control: TRPV1 control peptide, #ACC-030, alomone. kD – kiloDalton, MW – molecular weight marker.

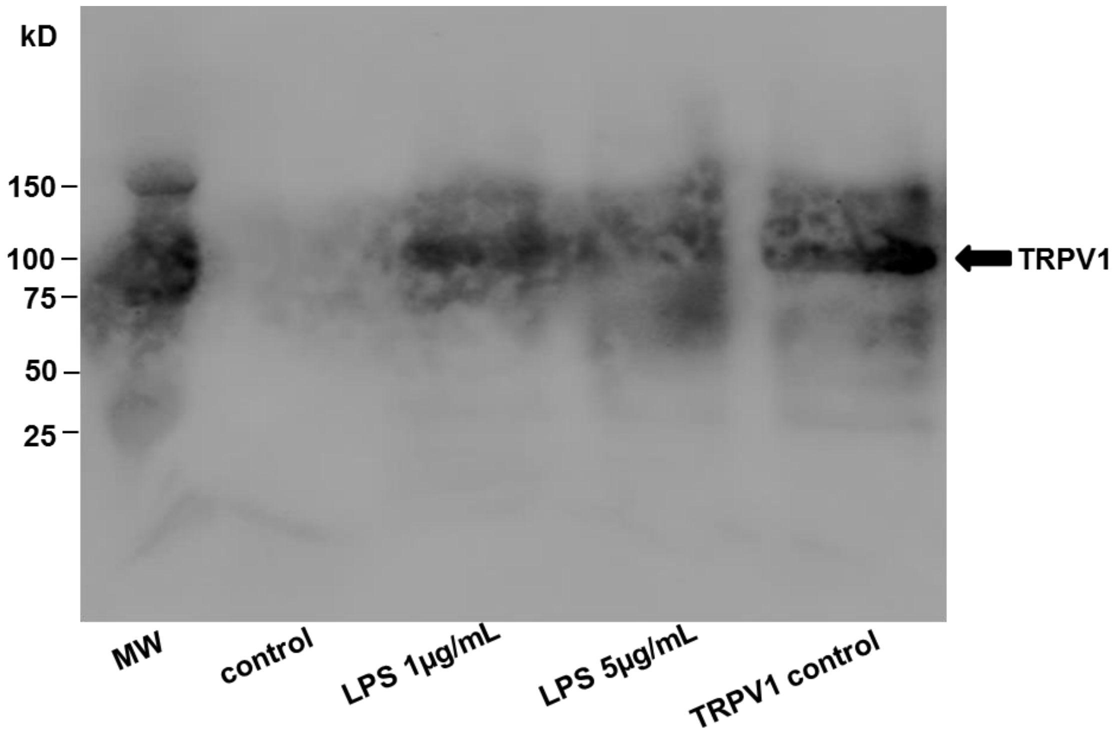

Supplement: Supplementary file 1 — Supplementary Figure 1. [file 41598_2021_93958_MOESM1_ESM.pdf]
